# Activatable Second Near-Infrared Fluorescent Probes: A New Accurate Diagnosis Strategy for Diseases

**DOI:** 10.3390/bios11110436

**Published:** 2021-11-02

**Authors:** Dong Li, Jie Pan, Shuyu Xu, Shiying Fu, Chengchao Chu, Gang Liu

**Affiliations:** State Key Laboratory of Molecular Vaccinology and Molecular Diagnostics, Center for Molecular Imaging, Translational Medicine School of Public Health, Xiamen University, Xiamen 361102, China; 32620201150737@stu.xmu.edu.cn (J.P.); xushuyu@stu.xmu.edu.cn (S.X.); 21620191152631@stu.xmu.edu.cn (S.F.); chuchengchao@xmu.edu.cn (C.C.)

**Keywords:** NIR-II fluorescent probes, activatable strategy, NIR-II fluorescence imaging, biomarker, biosensing

## Abstract

Recently, second near-infrared (NIR-II) fluorescent imaging has been widely applied in biomedical diagnosis, due to its high spatiotemporal resolution and deep tissue penetration. In contrast to the “always on” NIR-II fluorescent probes, the activatable NIR-II fluorescent probes have specific targeting to biological tissues, showing a higher imaging signal-to-background ratio and a lower detection limit. Therefore, it is of great significance to utilize disease-associated endogenous stimuli (such as pH values, enzyme existence, hypoxia condition and so on) to activate the NIR-II probes and achieve switchable fluorescent signals for specific deep bioimaging. This review introduces recent strategies and mechanisms for activatable NIR-II fluorescent probes and their applications in biosensing and bioimaging. Moreover, the potential challenges and perspectives of activatable NIR-II fluorescent probes are also discussed.

## 1. Introduction

Fluorescence imaging techniques are widely used in the fields of disease detection and diagnosis, surgical navigation, and drug delivery, due to their high sensitivity and noninvasiveness, and the absence of ionizing radiation [1,2,3]. According to different emission wavelengths, fluorescence imaging can be divided into three regions: the visible region (400–700 nm), the first near-infrared region (NIR-I, 700–900 nm), and the second near-infrared region (NIR-II, 1000–1700 nm) [4,5,6]. In comparison with NIR-I fluorescence imaging, NIR-II fluorescence imaging has the characteristics of less scattering, minimal tissue absorption, and low autofluorescence, which can produce deeper tissue penetration and a higher signal-to-background ratio (SBR) [7,8,9]. To date, varieties of NIR-II fluorescent probes, including benzobisthiadiazole (BBTD) dyes, semiconducting polymer nanoparticles (SPNPs), cyanine dyes, quantum dots (QDs), and single-wall carbon nanotubes (SWCNTs) have been developed for applications in NIR-II fluorescence imaging [10,11,12]. 

Recently, accurate in vivo diagnosis based on specific biomarkers produced by diseases has attracted extensive attention [13,14]. Specifically, the pathological microenvironment of diseased tissue is obviously different from that of normal tissue, and the occurrence and development of many diseases produce specific biomarkers [15,16,17]. For example, the pH value and the reactive oxygen species (ROS) concentration are abnormal in the tumor microenvironment [18,19,20], hepatotoxicity induced by drugs has an abnormal ONOO^−^ concentration [21], and liver injury induced by diabetes leads to viscosity changes [22]. Encouragingly, the specific biomarkers generated by the disease can be exploited to activate NIR-II fluorescence probes, realizing the accurate diagnosis of the underlying disease [23]. Compared with the “always on” types of NIR-II fluorescent probes, activatable NIR-II fluorescent probes can be specifically recognized by the target tissue and exhibit switchable fluorescence emission [13,14]. In fact, activatable NIR-II fluorescent probes cannot produce fluorescence, or produce only weak fluorescence; however, the special structure reacts with certain molecules existing in the microenvironment of the diseased tissue, thereby producing effective NIR-II fluorescence emission [24,25,26]. Consequently, the “off” state in normal tissues and the “on” state in the diseased tissues can significantly improve the sensitivity and resolution of the fluorescence imaging [27,28,29]. Therefore, it is of great significance to utilize disease-associated endogenous stimuli to specifically activate NIR-II fluorescent probes for highly sensitive and high-resolution bioimaging.

**Scheme 1:**
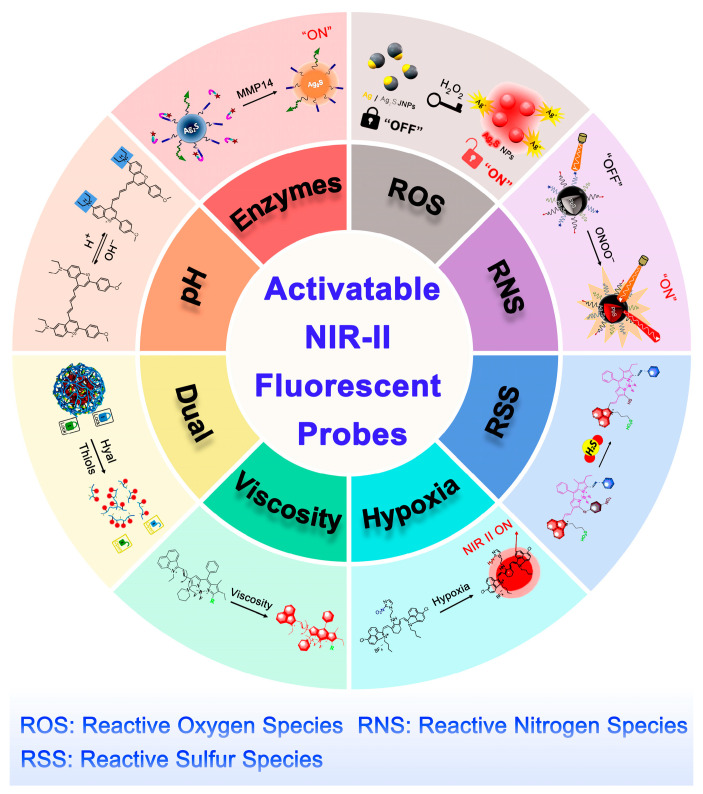
Schematic illustration of strategies and mechanisms for activatable NIR-II fluorescent probes. Image for pH: reproduced from publication of Wang, S. et al. with permission, Copyright 2019, Nature Publishing Group. Image for enzymes: reproduced from publication of Zhan, Y. et al. with permission, Copyright 2021, WILEY-VCH. Image for ROS: reproduced from publication of Zhang, X. et al. with permission, Copyright 2021, American Chemical Society. Image for RNS: reproduced from publication of Yang, X. et al. with permission, Copyright 2021, WILEY-VCH. Image for RSS: reproduced from publication of Dou, K. et al. with permission, Copyright 2021, American Chemical Society. Image for hypoxia: reproduced from publication of Meng, X. et al. with permission, Copyright 2018, Creative Commons. Image for viscosity: reproduced from publication of Dou, K. et al. with permission, Copyright 2021, American Chemical Society. Image for dual: reproduced from publication of Tang, Y. et al. with permission, Copyright 2018, WILEY-VCH.

Activatable NIR-II fluorescent probes (e.g., small organic molecular fluorophores, inorganic nanoparticles, and semiconducting polymer nanoparticles) for specific fluorescence imaging have made rapid progress. Here, recent strategies for activatable NIR-II fluorescent probes and their applications in biosensing and bioimaging are summarized (Scheme 1). In addition, according to the characteristics of highly expressed biomarkers in diseases, the activation mechanism is comprehensively analyzed and discussed. Finally, the prospects are also analyzed.

## 2. NIR-II Fluorescent Probes

### 2.1. Organic NIR-II Fluorescent Probes

**Figure 1:**
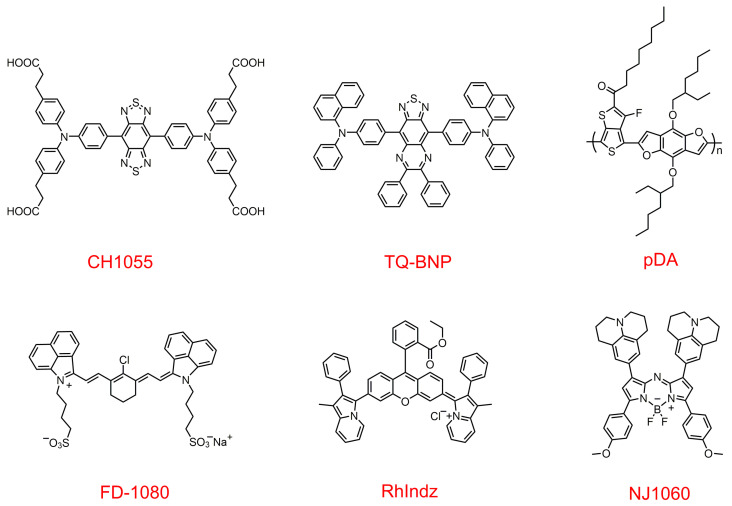
Chemical structures of CH1055, TQ-BNP, pDA, FD-1080, Rhlndz, and NJ1060.

Organic NIR-II fluorescent probes are applied in biological imaging for disease diagnosis, and mainly include BBTD dyes, aggregation-induced emission luminogens (AIEgens), SPNPs, cyanine dyes, rhodamine analogs, and boron dipyrromethenes (BODIPYs), with representative structures CH1055, TQ-BPN, poly (benzo [1,2-b:3,4-b′] difuran-alt-fluorothieno-[3,4-b] thiophene) (pDA), FD-1080, RhIndz, and NJ1060, respectively (Figure 1) [30,31]. Specifically, BBTD derivatives exhibit donor–acceptor–donor (D–A–D) characteristics with large Stokes shifts and high imaging quality [32]. In 2016, Antaris et al. reported the BBTD core structure CH1055 with fluorescence emission at 1055 nm, which outperformed the clinically used indocyanine green (ICG) for sentinel lymphatic imaging in the vicinity of mouse tumors [2]. In addition, a high enrichment of PEGylated CH1055 dye was observed in the deep tissues of mouse brain tumors at approximately 4 mm. Moreover, the tumor-to-normal tissue ratio of NIR-II imaging mediated by the CH1055 dye was 5 times higher than that of NIR-I imaging, enabling it to be used for in vivo imaging to guide tumor resection. In 2019, Zhou et al. developed two NIR-II fluorescent probes, CH1055-PEG-PT and CH1055-PEG-Affibody, which showed great potential in the fluorescence imaging of osteosarcoma and lung metastasis, respectively. In addition, CH1055-PEG-PT surpassed the imaging capability of computed tomography for a 143B tumor in vivo, and could therefore be used to guide surgical resection of 143B tumors. CH1055-PEG-Affibody could be used to visualize osteosarcoma and lung metastasis [33]. In contrast to the traditional supramolecular dyes, AIE molecules overcame the aggregation-caused quenching (ACQ) induced by intermolecular π–π stacking. Qi et al. reported a crab-shaped AIEgen, TQ-BPN, with fluorescence emission at 700–1200 nm for high-resolution microangiography and imaging in the NIR-II window [34]. The excellent imaging performance of TQ-BPN ensured visualization of the anatomy of high-depth brain capillaries (800 µm) with high spatial resolution (~3 µm). Therefore, TQ-BPN could be used to dynamically evaluate vascular diseases for the diagnosis of blood–brain barrier damage in the brain. Samanta et al. developed a highly bright, highly water-soluble aggregation-induced emission (AIE)-active two-photon (TP) (AIETP) NIR-II probe, which could form hydrophilic nanoparticles, AIETP NPs, with the polymer Pluronic F127. Encouragingly, AIETP NPs not only exhibited excellent cell permeability and biocompatibility but also exhibited good two-photon imaging properties in vivo. Moreover, due to the superior penetration depth (800 μm) and excellent spatial resolution (1.92 μm), AIETP NPs were applied to deep brain imaging in vivo [35]. SPNPs are organic semiconducting macromolecules whose backbone consists of alternating single and double bonds. Among these, pDA is a typical SPNP, with an emission wavelength of 1050 nm and a Stokes shift of approximately 400 nm, which can be used for vascular imaging in vivo [5]. Importantly, pDA has a frame rate of >25 frames per second, which can be applied to deep tissue and ultrafast imaging of mouse arterial blood flow. Cyanine dyes are based on a polymethylene skeleton and contain a unique extended conjugated system. Specifically, FD-1080 is a polymethine cyanine dye with an emission wavelength of 1080 nm, which can achieve noninvasive high-resolution angiography of deep-tissue brain and hindlimb vessels [36]. In addition to the abovementioned typical dyes, traditional NIR-I dyes modified with specific groups can achieve NIR-II fluorescence emission. RhIndz and NJ1060 are derivatives of rhodamine and BODIPY, respectively [37,38]. With the modification of special functional groups, both RhIndz and NJ1060 achieved NIR-II fluorescence emission. It is worth noting that organic NIR-II fluorescent probes show great potential in the application of surgical navigation. Zeng et al. designed a small-molecule fluorescent probe, H3-PEG2k, with excellent aqueous solubility, high brightness, and high photostability [39]. When H3-PEG2k was intravenously injected into rats with mammary carcinoma, a strong fluorescence signal could be observed within 8 h after injection. In addition, the tumor and the surrounding normal tissues could be clearly distinguished 8.5 h after injection. Encouragingly, H3-PEG2k was applied to rat mammary carcinoma imaging as well as image-guided tumor resection surgery. This research provided important guidance for the use of NIR-II fluorescence probes in clinical breast cancer imaging in vivo and in surgical navigation.

### 2.2. Inorganic NIR-II Fluorophores

Compared with organic NIR-II fluorescent probes, inorganic NIR-II fluorescent probes, including QDs, SWCNTs, and rare-earth-doped nanoparticles (RENPs) have excellent quantum yield and high stability [40,41]. Among the multiple NIR-II fluorescent probes reported thus far, QDs have attracted much attention due to their excellent fluorescence quantum yield [42]. Recently, a series of metal–sulfur QDs have been reported to possess excellent NIR-II fluorescence properties, including Ag_2_S, PbS, and ZnS [32]. Specifically, Ag_2_S QDs are widely used in preclinical research due to the excellent tunability of their optical properties, their excellent biocompatibility, and their low cytotoxicity. The NIR-II fluorescence imaging of Ag_2_S QDs has high spatial resolution (~40 μm) and can track angiogenesis mediated by tumors (2–3 mm in diameter) in vivo [43]. On the other hand, due to their narrow band gap, SWCNTs can generate a fluorescence emission spectrum with wavelengths from 1000 nm to 1800 nm, which can be used for in vivo NIR-II fluorescence imaging of tumor vessels in deep tissues [44]. Ghosh et al. reported a SWCNT modified with a SPARC binding peptide (SBP) and M13 phage, which can be used for the detection of tumor nodules on multiple abdominal viscera and the mesentery [45]. With the NIR-II fluorescence reflectance imaging system, SBP-M13-SWNT-mediated fluorescence imaging can detect tumors at a depth of 9.7–18.2 mm. Notably, compared with the fluorescent probes fluorescein isothiocyanate (FITC) and AlexaFluor750 dye (AF750), the tumor-to-muscle ratio of SBP-M13-SWNT was 5.5 ± 1.2, which was significantly higher than that of SBP-M13-FITC (0.96 ± 0.10) or SBP-M13-AF750 (3.1 ± 0.42). RENPs were also applied in NIR-II fluorescence imaging where rare earth (RE) metals were embedded in an inorganic crystalline host matrix (for example, NaYF_4_ or CaF_2_). In addition, RENPs possess an adjustable emission spectrum, long luminescence life and good photostability; hence, they are expected to replace traditional organic fluorescent materials for fluorescence imaging [7]. Lei et al. doped cerium ions (Ce^3+^) into the NaYbF_4_:Er^3+^ nanostructure and achieved fluorescence emission at 1550 nm [46], and further in vivo experiments showed that NaCeF_4_:Er/Yb could be used for deep-tissue NIR-II fluorescence imaging of mouse hind limbs. Xue et al. developed polyacrylic acid (PAA)-modified NaYF_4_:Gd/Yb/Er nanorods (PAA-NRs), which could be used for the visual detection of microscopic tumors via NIR-II fluorescence imaging. Notably, non-invasive high-resolution and highly spatial (down to 43.65 μm) NIR-II brain vasculature imaging was achieved using PAA-NRs [47]. In summary, a wide variety of NIR-II fluorescent probes have been prepared and have shown great potential in tumor imaging, deep-seated disease detection, surgical navigation, and therapeutic effect evaluation.

## 3. Activatable NIR-II Fluorescent Probes

Although NIR-II fluorescent probes have excellent optical properties for bioimaging, the “always on” fluorescent probes produce nonspecific signals in normal tissues, reducing the detection sensitivity [48]. In comparison with the “always on” fluorescent probes, activatable fluorescent probes achieve high specificity by increasing the target signal intensity and reducing the background signal [49]. According to the activation modes, these strategies mainly include eight categories: pH, enzymes, ROS, reactive nitrogen species (RNS), reactive sulfur species (RSS), hypoxia, viscosity and dual-responsive.

### 3.1. pH

**Figure 2:**
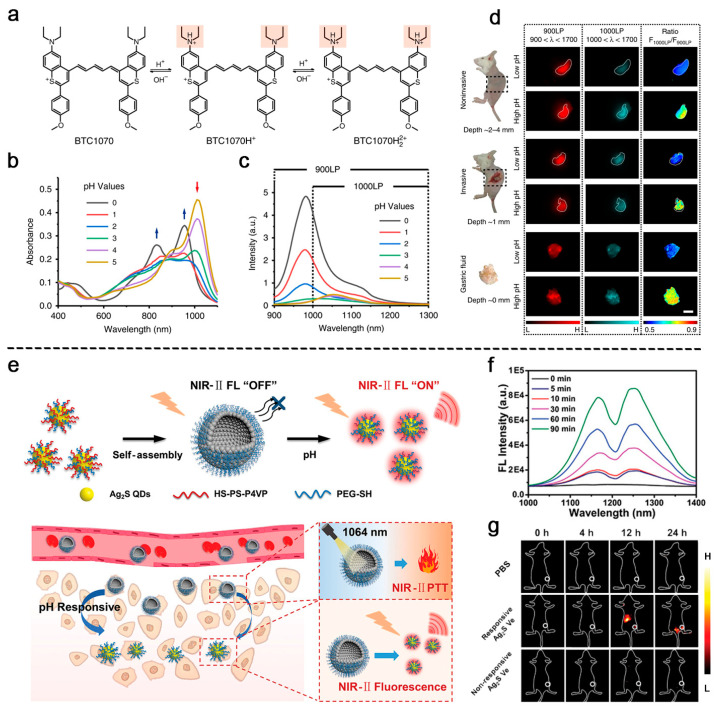
(**a**) Chemical structure of BTC1070 and its protonation mechanism. (**b**) Electronic absorption and (**c**) fluorescence spectra of BTC1070 in PBS at different pH conditions (excited at 808 nm). (**d**) Digital photographs of three imaging modes distinguished based on different tissue depths (left) and fluorescence imaging and ratiometric fluorescence imaging of BTC1070 in gastric juice at different pH under three imaging modes (right). Reproduced with permission [54]. Copyright 2019, Nature Publishing Group. (**e**) Schematic diagram of self-assembly process of Ag_2_S QDs into pH-activatable Ag_2_S Ve and the mechanism of pH-activatable Ag_2_S Ve for NIR-II fluorescence-imaging-guided photothermal cancer therapy in vivo. (**f**) Fluorescence spectra of pH-activatable Ag_2_S Ve triggered by low pH condition at different time points under the irradiation of 808 nm laser. (**g**) Time-dependent NIR-II fluorescence imaging of tumors in vivo after treatment with PBS and pH-activatable and non-activatable Ag_2_S Ve (10 mg/mL). Reproduced with permission [63]. Copyright 2021, WILEY-VCH.

pH plays an important role in the physiological homeostasis of the living body [50,51]. Abnormal pH values affect the physiological balance, which is related to the occurrence and development of a variety of diseases [13,52]. For example, the pH value in gastric juice can affect the activity of digestive enzymes in gastric juice and the utilization of oral drugs [53]. The pH value in gastric juice can be monitored using a pH-sensing fluorescent probe. In 2019, Wang et al. reported a benzothiopyrylium pentamethine cyanine substituted by diethylamino (BTC1070) probe with high-penetration NIR-II imaging properties, which exhibited superior pH-responsive properties (Figure 2a) [54]. Interestingly, when the pH value was reduced from 5 to 2, the maximum absorption peak at 1015 nm decreased and a new absorption peak at 600–900 nm appeared (Figure 2b). The values of pKa were 0.29 and 3.81 by Boltzmann curve fitting, which clearly indicated that BTC1070 had a double protonation feature. The protonation process led to the inhibition of the intramolecular charge transfer (ICT) effect, which realized the fluorescence response ratio (Figure 2c). In addition, mice were treated with simulated gastric juice of pH 1.3 or 2.5 to simulate the acidic environment of the human stomach. The fluorescence imaging of BTC1070 in gastric juices at different pH values was monitored by noninvasive ratiometric imaging at different tissue depths, and the results indicated that BTC1070 could be used for high-contrast fluorescence imaging of deep tissues, to noninvasively detect the pH value in gastric juice (Figure 2d). Overall, this investigation showed that pentamethine fluorophore could be used in pH-activated NIR-II fluorescence imaging for accurate detection of pH in gastric juice. 

Cancer is a heterogeneous disease which differs from normal tissue in morphology and growth mode [55,56,57]. The rapid proliferation of tumor cells leads to the characteristic changes in energy metabolism [58,59]. Tumor cells are vigorous with respect to energy metabolism, consuming large amounts of glucose and converting it to lactate, resulting in an increase in extracellular H^+^ concentration and a decrease in pH value [18,60,61]. Inorganic NIR-II fluorescent probes have a high degree of stability, which can be controlled by modifying the pH-responsive groups on their surfaces. In 2020, Ling et al. reported a NIR-II nanodrug system (FEAD1) for precise tumor theranostics via tumor acid activation, which was self-assembled by the Fmoc-His peptide, mercaptopropionic-modified Ag_2_S QDs (MPA-Ag_2_S QDs), NIR absorber A1094, and doxorubicin (DOX) [62]. The NIR-II fluorescence of FEAD1 was largely quenched, due to the Forster resonance energy transfer (FRET) between Ag_2_S QDs and A1094. However, under the acidic conditions of the tumor, the disassembly of FEAD1 by protonation of the imidazole groups led to a loss of FRET, enabling NIR-II fluorescence recovery. In vivo experiments showed that FEAD1 not only formed specific NIR-II fluorescence signals in the breast cancer sites of mice but also illuminated peritoneal metastatic tumor nodules over a long period, endowing this novel pH-activatable NIR-II fluorescent probe with excellent potential for clinical applications. In 2021, Liu et al. reported novel pH-sensitive nanovesicles assembled by thiolated polystyrene-co-poly(4-vinylpyridine)-modified Ag_2_S QDs (Ag_2_S Ve) for precisely activating NIR-II fluorescence imaging (Figure 2e) [63]. The pyridine group of 4-vinylpyridine was easily protonated at a lower pH value, demonstrating that Ag_2_S Ve had the capacity for pH responsiveness. As shown in Figure 2f, under acidic conditions the fluorescence intensity of Ag_2_S Ve gradually recovered in the range of 1000 to 1400 nm within 90 min. In vivo NIR-II fluorescence imaging indicated that Ag_2_S Ve showed obvious fluorescence in the tumor site at 12 and 24 h, while the control group showed no fluorescence, demonstrating excellent pH-responsive properties (Figure 2g). 

### 3.2. Enzymes

**Figure 3:**
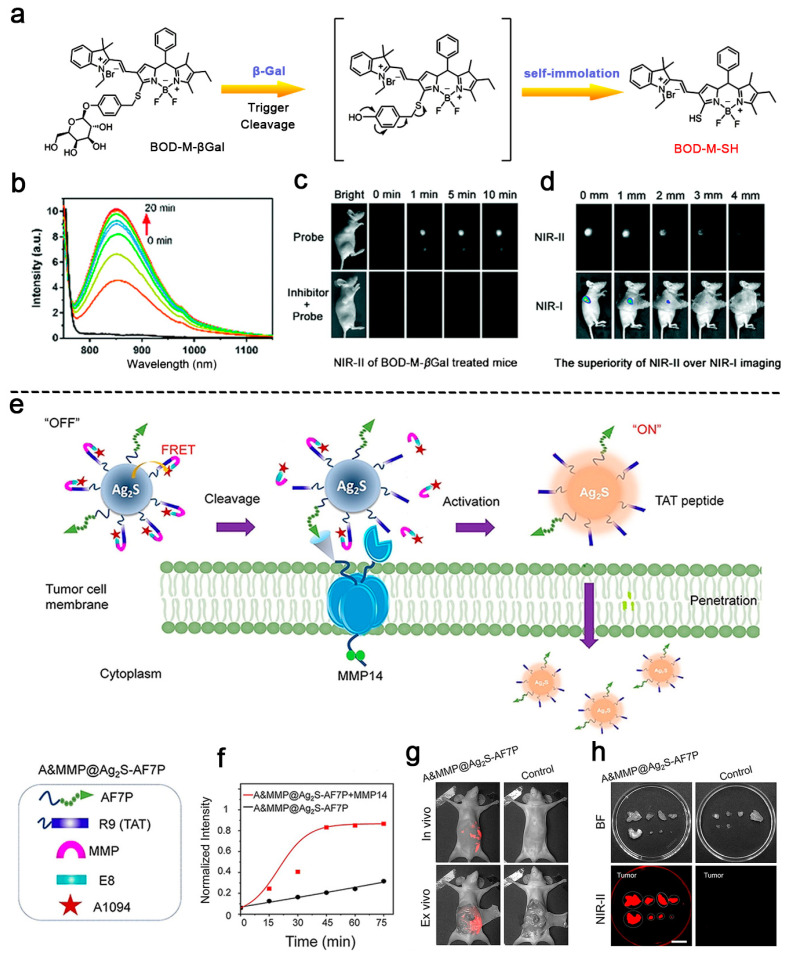
(**a**) Schematic illustration of the mechanism for β-Gal enzymatic activation process of BOD-M-βGal. (**b**) Time-dependent NIR-II fluorescence spectra of BOD-M-βGal upon addition of β-Gal in mixed aqueous solution. (**c**) NIR-II fluorescence imaging of mice at different time points after treatment with BOD-M-βGal and BOD-M-βGal + inhibitor D-galactose. (**d**) Comparison of NIR-II and NIR-I fluorescence imaging of mice after treatment with BOD-K-βGal and BOD-M-βGal in various deep-tissue environments. Reproduced with permission [72]. Copyright 2020, Royal Society of Chemistry. (**e**) Schematic diagram of A&MMP@Ag_2_S-AF7P for NB detection. (**f**) Validation of MMP14-mediated fluorescence recovery of A&MMP@Ag_2_S-AF7P. (**g**) NIR-II fluorescence and bright field (BF) imaging of peritoneal tumors in vivo and ex vivo after intraperitoneal injection of A&MMP@Ag_2_S-AF7P. (**h**) BF and NIR-II fluorescence imaging of resected tumor nodules (scale bar = 5 mm). Reproduced with permission [77]. Copyright 2021, WILEY-VCH.

Enzymes are an extremely important class of biocatalysts which are involved in a great number of biological reactions in living organisms, and abnormalities in their concentrations are often associated with the development of various diseases [64,65,66]. For example, β-galactosidase (β-Gal), an enzyme that can hydrolyze substances containing a β-glycoside bond, exists widely in living organisms [67,68]. An increase in the content of β-Gal in the human body usually induces the occurrence of ovarian cancer, and sensitive detection of β-Gal is therefore important for the early prediction and diagnosis of ovarian cancer [69,70,71]. Chen et al. developed an activatable galactose-modified BODIPY BOD-M-βGal NIR-II fluorescent probe (Figure 3a) [72]. The transition of BODIPY from NIR-I to NIR-II was achieved by providing elongation of the π-conjugation to BODIPY via a vinylene unit. As shown in Figure 3b, β-Gal enhanced the fluorescence of BOD-M-βGal significantly within 20 min, showing enzyme-responsive fluorescence emission in the NIR-II region. In vivo fluorescence imaging indicated that BOD-M-βGal achieved excellent imaging of tumor regions, while the additional inhibitor D-galactose significantly inhibited the fluorescence intensity, showing the specificity of the enzyme response (Figure 3c). Moreover, at a depth of 2 mm, the bright NIR-II signal of BOD-M-βGal could be observed, while the NIR-I fluorescence signal was barely detected (Figure 3d). 

Matrix metalloproteinase (MMP) is a type of matrix-degrading enzyme which plays an important role in atherosclerosis, rheumatoid arthritis, enteritis, cancer, and other inflammatory diseases [73,74,75]. Jeong et al. reported a protease-activatable QD (PA-NIR QD) NIR-II probe, which was obtained by combining a PbS/CdS/ZnS multishell fluorescent probe with activatable modulators (AcMs) [76]. When PA-NIR QD was loaded with the photosensitizer methylene blue (MB), the PA-NIR QD was quenched by photoinduced electron transfer (PET) and was activated specifically in the presence of MMP. After simultaneous incubation of MMP2 with PA-NIR QD, the fluorescence recovery efficiency of PA-NIR QD increased with an increase in MMP2. Furthermore, when the PA-NIR QD was injected in a tumor mouse model, the tumor area showed a stronger fluorescence signal than the normal tissue, indicating that MMP-activated PA-NIR QD achieved real-time, high-resolution fluorescence imaging. Recently, Zhan et al. reported the NIR-II fluorescent probe A&MMP@Ag_2_S-AF7P, specifically activated by the MMP14 enzyme for rapid diagnosis of neuroblastoma (NB) (Figure 3e) [77]. A&MMP@Ag_2_S-AF7P is modified by the MMP14-targeting peptide AF7P, polycationic peptide R9 and poly-anionic fragments E8, which are taken up by NB cells overexpressing MMP14, and FRET is disrupted, resulting in rapid activation of NIR-II fluorescence. As shown in Figure 3f, the extra MMP14 recovered the fluorescence of A&MMP@Ag_2_S-AF7P, and the fluorescent probe was lit up within 45 min, indicating that this enzymatic reaction was fast and efficient. In vitro and ex vivo experiments showed that A&MMP@Ag_2_S-AF7P could be activated by MMP14 to produce obvious NIR-II fluorescence signals (Figure 3g,h).

### 3.3. ROS

**Figure 4:**
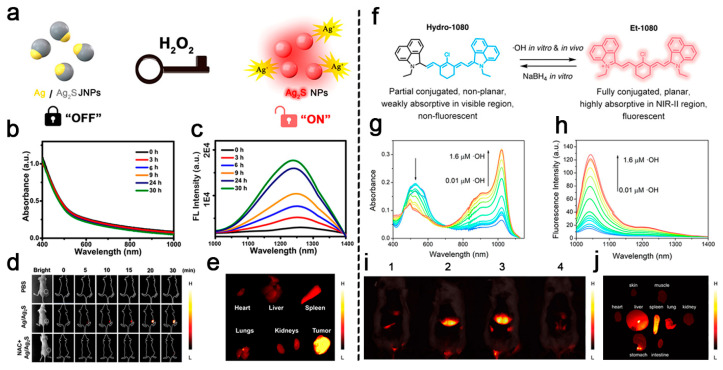
(**a**) Schematic representation of the mechanism of H_2_O_2_-activatable nanoprobe Ag/Ag_2_S JNPs for NIR-II fluorescence imaging in vivo. (**b**) Electronic absorption and (**c**) fluorescence spectra of Ag/Ag_2_S JNPs after treatment with H_2_O_2_ at different time points. (**d**) NIR-II fluorescence imaging of tumor-bearing mice at different time points after Ag/Ag_2_S JNP injection in vivo. (**e**) NIR-II fluorescence imaging of tumor and main organs excised from the Ag/Ag_2_S-treated mice group. Reproduced with permission [82]. Copyright 2021, American Chemical Society. (**f**) Chemical structures and mutual conversion processes of Hydro-1080 and Et-1080. (**g**) Electronic absorption and (**h**) fluorescence spectra of Hydro-1080 after reaction with different concentrations of ·OH. (**i**) NIR-IIa fluorescence imaging of mice injected first with different doses of APAP (0, 300, 500, 500 mg/kg) and then with Hydro-1080 (1 mM, 5 mL/kg). In addition, the fourth mouse in the picture was intraperitoneally pre-injected with ABT (100 mg/kg) 24 h before injecting APAP. 1: Hydro-1080; 2: APAP (300 mg/kg) + Hydro-1080; 3: APAP (500 mg/kg) + Hydro-1080; 4: APAP (500 mg/kg) + Hydro-1080 + ABT (100 mg/kg). (**j**) NIR-IIa fluorescence imaging of main organs and tissues of mice injected with APAP (500 mg/kg) and Hydro-1080. Reproduced with permission [88]. Copyright 2019, American Chemical Society.

ROS, mainly including hydrogen peroxide (H_2_O_2_), superoxide anion (O_2_^•^^−^), hypochlorite (ClO^−^), and hydroxyl radicals (·OH), can affect many physiological and pathological processes, such as cell apoptosis, cell signal transduction, and cancer [78,79,80]. Some investigations have shown that the H_2_O_2_ concentration in tumor tissue or at inflammatory sites was much higher than that in normal tissue [18,81]. In 2021, Zhang et al. reported a size-tunable Ag/Ag_2_S Janus NP (JNP) NIR-II fluorescent nanoprobe activated by endogenous H_2_O_2_ (Figure 4a) [82]. Plasma electron transfer led to the fluorescence quenching of Ag/Ag_2_S JNP; however, H_2_O_2_ could etch the surface Ag and thus activate its fluorescent property. As shown in Figure 4b,c, the Ag in Ag/Ag_2_S JNP was gradually etched in the presence of H_2_O_2_ within 30 h, resulting in a slight change in absorbance at 808 nm, while the NIR-II fluorescence intensity at 1250 nm was significantly enhanced. In vivo and ex vivo NIR-II fluorescence imaging indicated that mice injected with Ag/Ag_2_S JNP generated obvious fluorescence at the tumor site which could be inhibited by N-acetyl cysteine (NAC) (Figure 4d,e). However, ·OH is the ROS with the strongest oxidation activity in living organisms, and it can attack biological substrates such as biological proteins and DNA, causing severe oxidative damage [83,84]. Compared with conventional electron paramagnetic resonance (EPR), detection of ·OH, fluorescence imaging is noninvasive and highly sensitive, whereas NIR-II fluorescence imaging has deeper tissue penetrability and lower spontaneous background fluorescence [85,86,87]. In 2019, Feng et al. reported a NIR-II fluorescent probe, Hydro-1080, which could be activated by ·OH (Figure 4f) [88]. Due to the decrease in conjugation and coplanarity in Hydro-1080, there was no obvious NIR-II fluorescence intensity. After the formation of Et-1080 by ·OH oxidation, the fluorescence recovered with the recovery of the conjugation system. As shown in Figure 4g,h, with a concentration of ·OH from 0.01 to 1.60 μM, the absorption intensity at 1021 nm was enhanced, and the fluorescence intensity at 1044 nm was also enhanced, showing the responsiveness of Hydro-1080 to ·OH. The production of ·OH in mouse liver could be induced by injecting acetaminophen (APAP) into mice. After different doses of APAP were injected into mice, and they were then injected with Hydro-1080, the mice showed a dose-dependent fluorescence increase in NIR-IIa (1300–1400 nm), and inhibitor 1-aminobenzotriazole (ABT) significantly inhibited the process (Figure 4i,j).

**Figure 5:**
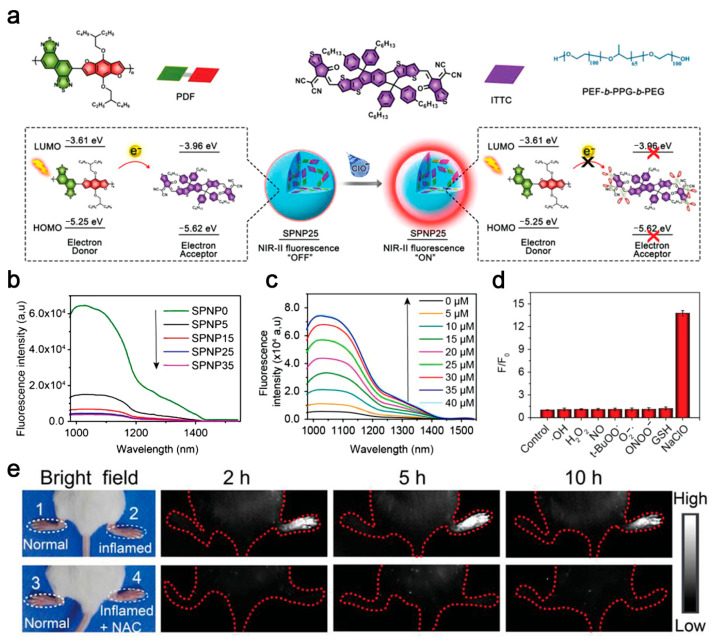
(**a**) Chemical structures of PDF, ITTC, and PEG-b-PPG-b-PEG, and the mechanism of nanoprobe SPNP25 for endogenous ClO^−^ detection. (**b**) Fluorescence intensity of SPNPs doped with different amounts of ITTC. (**c**) Fluorescence spectra of the nanoprobe SPNP25 after reaction with various concentrations of ClO^−^. (**d**) Fluorescence intensity ratios of nanoprobe SPNP25 toward ClO^−^ (40 μM) and various biochemically related analytes (100 μM). (**e**) Time-dependent fluorescence imaging in the inflammatory and normal sites of LPS-pretreated and LPS/NAC-treated mice after injection of SPNP25. Reproduced with permission [95]. Copyright 2019, WILEY-VCH.

Hypochlorous acid (HClO) is a highly oxidation-active oxo-acid of chlorine, which is endogenously produced from chloride ions and H_2_O_2_ catalyzed by myeloperoxidase (MPO) in neutrophils [89,90,91]. As a signal molecule, HClO is involved in regulating a variety of physiological processes [89,90,91]. However, excessive production of HClO will cause tissue damage and the formation of a variety of diseases, including cancer, arthritis, and lymphadenitis [92,93]. Ge et al. synthesized a novel NIR-II organic fluorescent probe, SETT, which could be activated by HClO-specific oxidation and showed a highly sensitive response [94]. Furthermore, SETT was loaded on the surface of down-conversion nanoparticles (DCNP) doped with Er^3+^, obtaining the NIR-II ratiometric nanoprobe DCNP@SeTT. Interestingly, upon light irradiation at a wavelength of 980 nm, the fluorescence intensity of SETT at 1150 nm decreased with an increasing concentration of HClO, while that of DCNP at 1550 nm was unchanged. Therefore, the ratiometric fluorescence signal (I1150 nm/I1550 nm) was linearly correlated with the concentration of HClO. In addition, the DCNP@SeTT probe could be used for HClO-responsive NIR-II fluorescence imaging of rabbit osteoarthritis and mouse tumors. Tang et al. reported that a non-fullerene acceptor (ITTC) was blended with a semiconducting polymer donor (PDF) to construct a NIR-II fluorescent probe (SPNPs) that could be activated by ClO^−^ (Figure 5a) [95]. Upon close contact of ITTC and PDF, the donor–acceptor interaction caused PET to quench the fluorescence of PDF. As shown in Figure 5b, with an increase in ITTC content, the NIR-II fluorescence quenching degree of SPNPs also increased. Encouragingly, ClO^−^ could oxidize and degrade ITTC, allowing the fluorescence of SPNP25 (25% doping amount) to recover, while other reactive oxygen species and reducing species could not produce this change, indicating the specificity of this activation process (Figure 5c,d). After intravenous injection of SPNP25, the fluorescence at the inflammatory sites of the mice was significantly enhanced, while there was no fluorescence at the normal sites (Figure 5e). It is worth noting that NAC significantly inhibited fluorescence at sites of inflammation in mice.

### 3.4. RNS

RNS are highly reactive biological oxidation species containing nitrogen, mainly including nitric oxide (NO), peroxynitrite (ONOO^−^), and S-nitrosothiols (RSNO) [21,96]. In common with ROS, RNS play an important role in oxidation processes under physiological conditions which are related to oxidative stress, brain diseases, cancer, and inflammation [97,98,99]. In fact, ONOO^−^ is not only related to the nitration of proteins in immune cells in tumors, affecting the immunosuppression of tumors, but is also related to the occurrence and development of brain injury [100,101]. 

**Figure 6:**
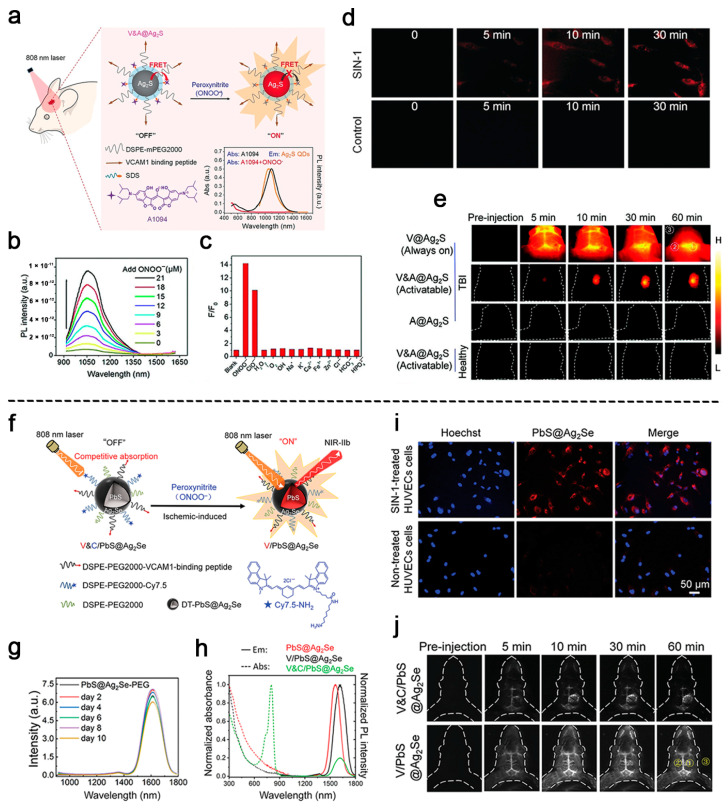
(**a**) Schematic diagram of the preparation process of the NIR-II nanoprobe V&A@Ag_2_S and the mechanism of detecting ONOO^−^ in vivo. (**b**) Photoluminescence spectra of V&A@Ag_2_S (50 μg/mL) after reaction with the different concentrations of ONOO^−^. (**c**) Fluorescence intensity ratios of V&A@Ag_2_S (10 μg/mL) upon the addition of different ions and RNS/ROS analytes (20 μM). (**d**) Fluorescence microscopy images of HUVECs after incubation with V&A@Ag_2_S in the presence and absence of SIN-1. (**e**) Time-dependent fluorescence imaging in TBI and healthy mice after different treatments. Reproduced with permission [105]. Copyright 2020, WILEY-VCH. (**f**) Schematic diagram of the design strategy of the NIR-II nanoprobe V&C/PbS@Ag_2_Se and the mechanism of detecting ONOO^−^ in ischemic stroke model mice. (**g**) Time-dependent fluorescence spectra of PbS@Ag_2_Se-PEG QDs in PBS of pH 7.4. (**h**) Electronic absorption and fluorescence spectra of V&C/PbS@Ag_2_Se, V/PbS@Ag_2_Se, and PbS@Ag_2_Se. (**i**) Fluorescence microscopy images of HUVECs after incubation with V&C/PbS@Ag_2_Se in the presence and absence of SIN-1. (**j**) Time-dependent NIR-II fluorescence imaging of early ischemic stroke model mice after treatment with V&C/PbS@Ag_2_Se and V/PbS@Ag_2_Se. Reproduced with permission [109]. Copyright 2021, WILEY-VCH.

Traumatic brain injury (TBI) is highly likely to cause disability and death in humans unless it can be easily diagnosed at an early stage [102]. Currently, clinical diagnosis of TBI is mainly achieved using magnetic resonance imaging (MRI) and computed tomography (CT), but these are difficult to use for real-time early diagnosis of TBI. As reported, ONOO^−^ is a biomarker of early TBI which is accompanied by the occurrence and development of TBI [103,104]. Therefore, the occurrence of early TBI can be diagnosed by monitoring the biomarker ONOO^−^. In 2020, Li et al. reported an activated NIR-II fluorescent probe (V&A@Ag_2_S) composed of a targeting group (VCAM1 binding peptide), an A1094 chromophore NIR absorber, and Ag_2_S QD (Figure 6a) [105]. Specifically, there is a large overlap between the NIR-II fluorescence emission spectrum of Ag_2_S QD and the absorption spectrum of A1094, leaving the fluorescence of V&A@Ag_2_S in the “off” state. However, when ONOO^−^ was present, A1094 was oxidized and its absorption peak disappeared, resulting in the NIR-II fluorescence signal of V&A@Ag_2_S being turned “on”. In addition, the VCAM1 binding peptide enabled V&A@Ag_2_S to target inflamed endothelium expressing VCAM1. As shown in Figure 6b, ONOO^−^ enhanced the fluorescence intensity of V&A@Ag_2_S at 1050 nm, and the magnitude of the enhancement had a linear relationship with the concentration of ONOO^−^. Apart from ONOO^−^/ClO^−^, other common anions and cations could not activate V&A@Ag_2_S, showing the specificity of this activation mode (Figure 6c). Encouragingly, 3-morpholinosydnonimine hydrochloride (SIN-1) was used to induce ONOO^−^ production by the cells, and cell experiments showed that in the presence of ONOO^−^ an obvious NIR-II fluorescence signal appeared after V&A@Ag_2_S uptake by human umbilical vein endothelial cells (HUVECs), whereas the control group did not exhibit this process (Figure 6d). In vivo experiments on TBI mice showed that V&A@Ag_2_S could be activated by ONOO^−^, showing excellent NIR-II fluorescence imaging. In the control group, the “always on” V@Ag_2_S probe was distributed over the whole brain region without specificity, while A@Ag_2_S exhibited no obvious fluorescence signal. Notably, V&A@Ag_2_S could not produce an obvious fluorescence signal in healthy mice, indicating that the “turned on” probe showed excellent specificity in biosensor and imaging applications (Figure 6e).

Ischemic stroke is also a brain disease, which is highly likely to cause death and disability in humans if it cannot be easily diagnosed at an early stage [106,107]. Similarly, ONOO^−^ is a biomarker of ischemic stroke and therefore provides the possibility of early diagnosis of the disease [103,108]. In 2021, Yang et al. reported a V&C/PbS@Ag_2_Se nanoprobe modified by VCAM1 binding peptide and Cy7.5 fluorophores, which could be activated by ONOO^−^ to produce obvious NIR-II fluorescence intensity (Figure 6f) [109]. Meanwhile, the VCAM1 binding peptide provided V&C/PbS@Ag_2_Se with the ability to target ischemic stroke regions, while the Cy7.5 fluorophores and PbS@Ag_2_Se QDs engendered competitive absorption to turn off the NIR-II fluorescence. As shown in Figure 6g, the stability of PbS modified by Ag_2_Se was significantly improved, and no significant change in fluorescence occurred within 10 days. Interestingly, both PbS@Ag_2_Se and V/PbS@Ag_2_Se exhibited obvious fluorescence at 1616 nm, while the fluorescence of V&C/PbS@Ag_2_Se was quenched (Figure 6h). Absorption-competition-induced emission (ACIE) was first proposed by Zhang et al. Specifically, organic dye acted as the absorption-competition acceptor of the fluorescent probe, significantly inhibiting the fluorescence emission of the fluorescent probe. ACIE technology could be used not only for monitoring drug release but also for in situ NIR sensing of biomarkers [110]. In addition, after HUVECs were incubated with SIN-1 and V&C/PbS@Ag_2_Se, the cells showed an obvious NIR-II fluorescence signal; conversely, in the absence of SIN-1 no NIR-II fluorescence signal was observed (Figure 6i). Furthermore, in vivo experiments showed that ONOO^−^ activated V&C/PbS@Ag_2_Se and lit up its NIR-II fluorescent signal (Figure 6j).

In contrast to ROS, NO is a widely existing messenger molecule in the human body which plays an important role in cardiovascular, cerebrovascular, immune system and nervous system processes [111]. It is noteworthy that NO was considered as a biomarker of drug-induced hepatotoxicity [112,113]. Iverson et al. reported a NIR-II fluorescent probe, PEG-(AAAT)7-SWNTs, produced by covering the NO-sensitive DNA oligonucleotide ds(AAAT)7 on the surface of SWCNTs [114]. The PEG-(AAAT)7-SWNTs nanoprobe could be used to investigate the production of NO by inflammation in vivo. In 2019, Tang et al. reported an organic semiconductor nanoprobe (AOSNP) activated by NO for monitoring drug-induced hepatotoxicity, which was obtained by the amidation reaction of the NO-sensitive organic semiconducting group (FTBD) and poly (styrene-co-maleic anhydride) (PSMA) polymer [115]. Notably, in the presence of NO, FTBD could transform its receptor unit (benzo[c] [1,2,5] thiadiazole-5,6-diamine) into benzotriazole derivatives, realizing the conversion of fluorescence from NIR-I to NIR-II. NO significantly increased the NIR-II fluorescence intensity of AOSNP and changed the color of the AOSNP solution. However, other reactive oxygen species (H_2_O_2_, ·OH, O_2_^•−^, and ONOO^−^) and reducing substances (glutathione and GSH) could not enhance the fluorescence of AOSNP, indicating the specificity of the activation mode. After co-incubation with AOSNP and HepG2 hepatoma cells, the cells showed an obvious NIR-II fluorescence signal, and the additional inhibitor NAC significantly inhibited this process. Interestingly, APAP induced a large amount of NO to produce hepatotoxicity in mice. In vivo fluorescence imaging indicated that AOSNP could be activated by NO generated from APAP-treated mice to produce obvious NIR-II fluorescence signals, while this process could be inhibited by inhibitor NAC.

### 3.5. RSS

RSS mainly include thiols (GSH and Cys), H_2_S, and persulfides (R-S-SH/H_2_S_2_) [116,117]. In fact, the level of intracellular GSH is higher than that of extracellular GSH, and the concentration of GSH in tumor tissue is also higher than that in normal tissue [26,118]. Li et al. reported a Ln^3+^-doped nanoparticles (LnNPs) probe using the large spectral overlap between the absorption spectrum of Ln^3+^ and the emission spectrum of heptamethine cyanine, which could be activated by GSH for high-resolution biological imaging [119]. In contrast to ROS and RNS, RSS not only played the role of oxidation, but also played the role of reduction. H_2_S is both a special biological signaling molecule and an important biomarker for early cancer [120,121,122]. In 2018, Shi et al. reported a H_2_S-activatable nanodrug (Nano-PT) for NIR-II fluorescence imaging of colorectal cancer (CRC) [116]. Nano-PT is a monochlorinated BODIPY derivative which produces a nucleophilic reaction with H_2_S. Interestingly, Nano-PT not only produced activatable NIR-II fluorescence emission, but also produced high-efficiency NIR absorption, which realized NIR-II-fluorescence-guided PTT. Therefore, PTT mediated by Nano-PT achieved highly specific CRC treatment guided by NIR-II fluorescence imaging, promoting the development of precision medicine.

**Figure 7:**
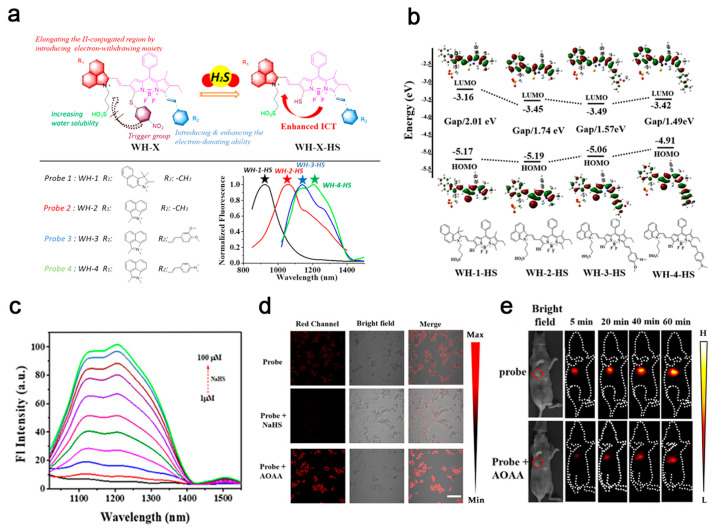
(**a**) Schematic illustration of the construction of several H_2_S-activatable NIR-II fluorescence probes WH-X (WH-1, WH-2, WH-3, and WH-4). (**b**) Theoretical calculations used to verify the design strategy. The HOMO and LUMO energy levels of WH-X-HS were computed. (**c**) Fluorescence spectra of WH-4 in the presence of different concentrations of NaHS under the irradiation of 980 nm laser. (**d**) Intracellular fluorescence imaging of HCT-116 cells incubated with WH-3, WH-3 + NaHS, and WH-3 + AOAA. (**e**) NIR-II fluorescence imaging of tumor-bearing mice at various time points after treatment with WH-3 and WH-3 + AOAA. Reproduced with permission [121]. Copyright 2021, American Chemical Society.

In 2021, Liu et al. designed and synthesized a series of H_2_S-activated NIR-II fluorescence probes, WH-X (WH-1, WH-2, WH-3, and WH-4), which were modified by a 4-nitrothiophenol fluorescence quencher (Figure 7a) [121]. The maximum emission wavelength of WH-3 was 1205 nm, which was superior to the other three compounds, and it showed excellent NIR-II fluorescence characteristics. Theoretical calculations showed that substitution of the WH-2-HS acceptor successfully reduced the gap between the highest occupied molecular orbital (HOMO) and the lowest unoccupied molecular orbital (LUMO) (Figure 7b). By extending the conjugation region in WH-3-HS and WH-4-HS, the ICT process was strengthened, leading to the gap being narrowed further. As shown in Figure 7c, WH-3 showed a concentration-dependent fluorescence enhancement in the presence of NaHS. Using H_2_S-overexpressing HCT-116 colon cancer cells as a model, intracellular fluorescence imaging also showed that NaHS significantly depleted WH-3, whereas the inhibitor aminooxyacetic acid (AOAA) inhibited this depletion (Figure 7d). In vivo experiments showed that WH-3 produced an NIR-II fluorescence signal at the tumor site, while the inhibitor AOAA inhibited this process, indicating that WH-3 could be activated by H_2_S (Figure 7e).

### 3.6. Hypoxia

**Figure 8:**
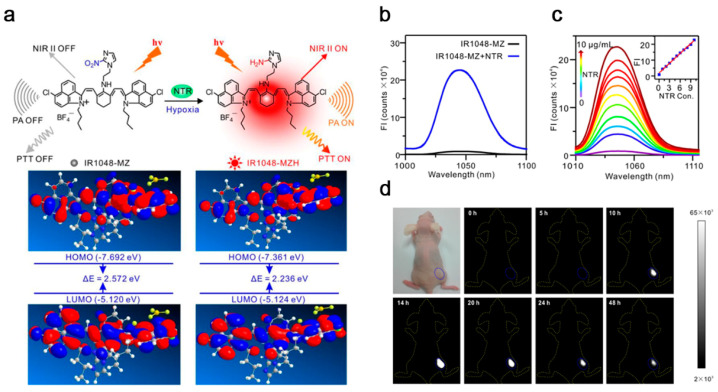
(**a**) Schematic diagram of the catalyzed mechanism of IR1048-MZ for hypoxia-activated NIR-II/PA tumor imaging. HOMO and LUMO energy levels of IR1048-MZ and IR1048-MZH were obtained by theoretical calculation. (**b**) Fluorescence spectra of IR1048-MZ with and without the addition of NTR. (**c**) NIR-II fluorescence spectra of IR1048-MZ (5 μg/mL) in response to various concentrations of NTR. (**d**) Time-dependent NIR-II fluorescence imaging of tumor-bearing mice after injection of IR1048-MZ (40 μg/mL, 200 μL). Reproduced with permission [126]. Copyright 2018, Creative Commons.

Hypoxia is an important characteristic of solid tumors which is related to tumor migration, invasion, and deterioration [123,124]. The occurrence of hypoxia greatly limits the therapeutic efficacy of chemotherapy, photodynamic therapy (PDT), and sonodynamic therapy (SDT) [125,126]. Therefore, it is significant to design hypoxia-activated nanomaterials for the diagnosis and treatment of tumors [127]. Meng et al. coupled IR-1048 dye and 2-(2-nitroimidazolyl) ethylamine, MZ, to develop a novel dye IR1048-MZ, achieving hypoxia-activated NIR-II/PA tumor-imaging-guided PTT (Figure 8a) [126]. Theoretical calculations showed that the HOMO and LUMO of IR1048-MZH were lower than those of IR1048-MZ, indicating enhanced fluorescence emission intensity. Nitroreductase (NTR) overexpressed by hypoxic tumor tissue reduced the nitro group of IR1048-MZ to an amine group and activated the NIR-II fluorescence signal of IR1048-MZ. As shown in Figure 8b, the fluorescence of the IR1048-MZ probe at the maximum emission wavelength (1046 nm) was suppressed, whereas it was significantly enhanced in the presence of NTR. In addition, with an increase in NTR concentration, the NIR-II fluorescence intensity also increased, and this change showed a good linear relationship (Figure 8c). Moreover, in vivo imaging showed that IR1048-MZ resulted in the A549 tumor of nude mice producing an obvious fluorescence signal, while no obvious signal was found in other parts (Figure 8d). The maximum value of the tumor background was 30, indicating that IR1048-MZ was an excellent probe for hypoxia without background.

### 3.7. Viscosity

**Figure 9:**
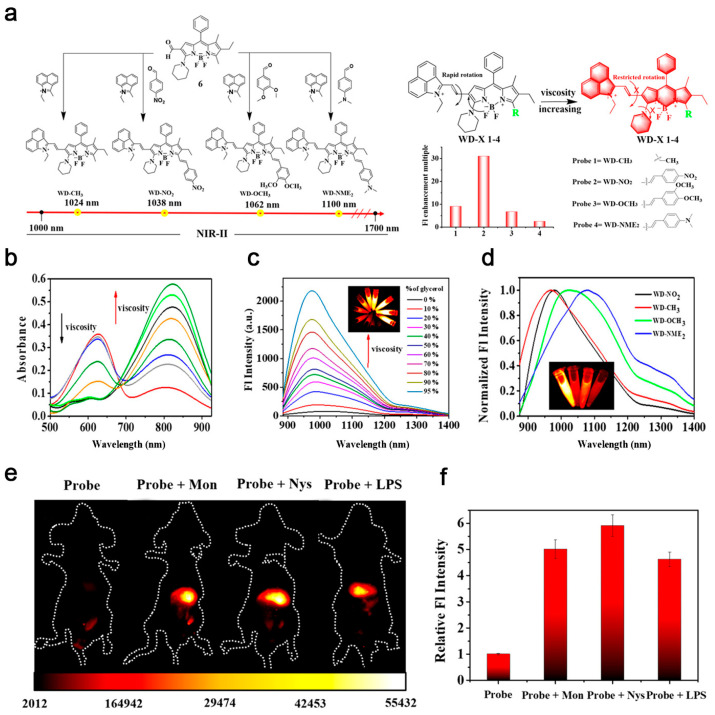
(**a**) Schematic illustration of the design strategy of viscosity-activatable fluorescence probes WD-X (WD-CH_3_, WD-NO_2_, WD-OCH_3_, and WD-NME_2_). Chemical structures and synthetic routes of WD-X (left) and mechanism of WD-X for viscosity-activatable NIR-II fluorescence imaging (right). (**b**) Electronic absorption and (**c**) fluorescence spectra of WD-NO_2_ (20 μM) in the ethanol-glycerol system with different viscosities. Inset shows fluorescence images of WD-NO_2_ in the ethanol-glycerol system with different glycerol ratios. (**d**) Normalized NIR-II fluorescence spectra of WD-X (WD-NO_2_, WD-CH_3_, WD-OCH_3_, and WD-NME_2_) in the system with 95% glycerol. Inset shows the corresponding fluorescence images under the irradiation of 808 nm (40 mW/cm^2^) laser. (**e**) Fluorescence imaging and (**f**) normalized relative fluorescence intensity of mice after treatment with WD-NO_2_, WD-NO_2_ + Mon, WD-NO_2_ + Nys, and WD-NO_2_ + LPS. Reproduced with permission [131]. Copyright 2020, American Chemical Society.

Viscosity is a major factor in the microenvironment of the living body which is related to various physiological and pathological processes [22,128]. Viscosity abnormalities are associated with many diseases, such as diabetes and Alzheimer’s disease [129,130]. Consequently, a viscosity-activated NIR-II fluorescent probe can be used to analyze and detect the viscosity level of organisms to further explore the occurrence and development of related diseases. In 2020, Dou et al. developed a series of viscosity-activated NIR-II fluorescent probes using BODIPY derivatives modified by 1-ethyl-2-methyl-benz[c,d] iodolium salt as a precursor (Figure 9a) [131]. It is worth emphasizing that the substituents of WD-OCH_3_ and WD-NME_2_ were strong electron-donating groups and exhibited longer NIR-II fluorescence emission wavelengths (Figure 9b). Interestingly, in the ethanol–glycerol system with different viscosities, the intensities of WD-NO_2_ at the maximum absorption wavelength and the maximum emission wavelength increased with an increase in viscosity, while the other three molecules also showed similar properties (Figure 9c,d). Briefly, at low viscosity, the conjugated structure was destroyed and caused an increase in nonradiative energy consumption, thereby leading to the weakening of the fluorescence intensity. However, at high viscosity, intramolecular rotation was restricted, causing fluorescence recovery. As shown in Figure 9e,f, monensin (Mon), nystatin (Nys), and lipopolysaccharide (LPS) could change the viscosity in mice. In vivo fluorescence imaging showed that the livers of mice treated with Mon, Nys, and LPS, which received intraperitoneal injections of WD-NO_2_, exhibited significant fluorescence intensity, indicating that WD-NO_2_ is an effective tool for studying the change of viscosity in vivo.

### 3.8. Dual-Responsive

**Figure 10:**
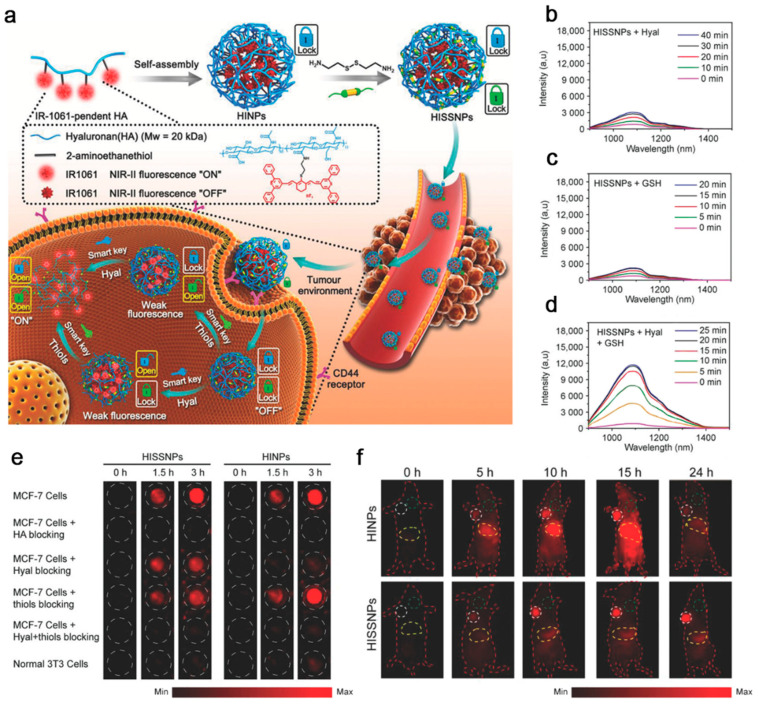
(**a**) Schematic diagram of the synthetic route of dual-lock-and-key-controlled NIR-II fluorescence probe HISSNPs and its corresponding mechanism for tumor-specific imaging. Time-dependent fluorescence (excited at 808 nm) spectra of HISSNPs incubated with (**b**) Hyal, (**c**) GSH, and (**d**) Hyal and GSH in PBS. (**e**) NIR-II fluorescence imaging of 3T3 cells and MCF-7 breast cancer cells at the time points of 0 h, 1.5 h, and 3 h after different treatments. (**f**) Time-dependent NIR-II fluorescence imaging of MCF-7 cancer xenografts model mice after injection with HISSNPs and HINPs (35 mg/kg). White circle: tumor site; yellow circle: abdominal liver site; green circle: muscle (refers to normal tissue). Reproduced with permission [139]. Copyright 2018, WILEY-VCH.

A single activation method cannot effectively deal with the complex and dynamic biological microenvironment, resulting in the phenomenon of nonspecific activation and even false-positive results [132,133]. The utilization of two activation methods in the above methods (pH, enzyme, redox, etc.) to activate the NIR-II fluorescence probe greatly improves the specificity of fluorescence imaging, which can effectively avoid the occurrence of false-positive results [134,135,136]. Zhang and co-workers developed a dual-activatable theranostic nanoprobe (DATN) which could output dual signals under the double stimulation of NO/acidity in inflammation-related tumors for photoacoustic and photothermal imaging in vivo [137]. DATN showed a higher photoacoustic signal under dual activation, which was 132 times that of acidity alone and 9.8 times that of NO. Under single-factor stimulation, DATN increased by about 6 °C. while under dual-factor stimulation it increased by about 27.3 °C, exhibiting the superiority of dual activation. In addition, in the tumor microenvironment, high levels of GSH (1–15 mM) and low pH (pH = 5.0–6.8) acted as a “dual key” to activate the photosensitizers. Teng et al. reported a photosensitizer, BIBCl-PAE NP, which underwent protonation under acidic conditions, further promoting the reaction of GSH with BIBCl-PAE NP to generate water-soluble BIBSG for PDT [138]. In vitro studies revealed that BIBCl-PAE NP could distinguish normal and cancer cells in cell imaging, while also exhibiting excellent PDT ability. In vivo experiments also showed that BIBCl-PAE NP was rapidly enriched in the tumor site, while specifically “lighting up” the tumor site, showing irreversible therapeutic activity with less effect on normal tissues. Tang et al. utilized NIR-II cyanine dye (IR-1061) to covalently connect hyaluronic acid (HA) and synthesized IR-1061-pendent HA polymers, which could self-assemble into single-lock-and-key-controlled HINPs in water, and then be cross-linked on the surface of HINPs through disulfide to form dual-lock-and-key-controlled HISSNPs (Figure 10a) [139]. HA and disulfide formed the “double locks” to lock HISSNPs in a quenched state, while the overexpressed hyaluronidase (Hyal) and GSH in the tumor microenvironment acted as “dual smart keys” to break HA chains and disulfide bonds enabled the fluorescence recovery of IR-1061. As shown in Figure 10b–d, when a “single smart key” (Hyal or GSH) existed, the fluorescence of HISSNPs was increased by about 3.4 times and 2.9 times, respectively, while when Hyal and GSH existed together, the fluorescence of HISSNPs was increased by about 13.3 times. In vitro cell experiments showed that MCF-7 breast cancer cells produced obvious fluorescence after uptake of HISSNPs, whereas normal cells did not show the phenomenon (Figure 10e). As we know, 6-O-palmitoyl-l-ascorbic acid (an inhibitor of Hyal) and N-ethylmalemide (a thiol scavenger) could significantly inhibit the activation process, while 6-O-palmitoyl-l-ascorbic acid could inhibit the activity of HINPs. In vivo fluorescence imaging indicated that mice injected with HISSNPs generated obvious NIR-II fluorescence at tumor sites with a higher sensitivity than that of HINPs, indicating the sensitivity and specificity of the dual lock and key (Figure 10f). Notably, although the dual-responsive NIR-II fluorescent probe showed excellent specificity, activatable NIR-II fluorescent probes with single activation methods are still in their infancy, and activatable NIR-II fluorescent probes with two activation methods have rarely been reported.

## 4. Summary and Outlook

Compared with traditional NIR-I fluorescence imaging, NIR-II fluorescence imaging has a deeper tissue penetration depth and a higher SBR. In recent years, a great number of NIR-II fluorescent materials, including SWCNTs, QDs, RENPs, BBTDs, and cyanine dyes were reported to have excellent photophysical and photochemical properties. However, these “always on” fluorescent probes lacked selectivity, showing a low SBR and poor detection sensitivity. On the other hand, the activatable NIR-II fluorescent probe is in an “off” state in normal tissues, while in diseased tissues it can be activated by a specific biomarker to present an “on” state. According to the type of activation mode, these strategies mainly include eight categories: pH, enzyme, ROS, RNS, RSS, hypoxia, viscosity, and dual-responsive. Specifically, the activation mechanisms were mainly the consumption of an inhibitor and changes in the functional group, charge, and conformation. These activatable NIR-II fluorescent probes and their activation strategies and mechanisms are summarized in Table 1.

Although activatable NIR-II fluorescent probes have made encouraging progress, they still face some challenges, mainly reflected in the following. (1) False-positive results. This is mainly due to the limited difference in the concentration of biomarkers in diseased tissues and normal tissues, which may cause false-positive results in NIR-II fluorescence imaging. For example, NB highly expresses MMP; however, many normal cells also produce MMP. It is necessary to consider how to avoid false-positive results affecting the diagnosis of the disease. On the one hand, the probe can be activated in a dual-responsive manner, improving the specificity of imaging. On the other hand, the occurrence of false-positive results is maximally avoided by setting the limit of detection. (2) Delivery of the fluorescent probe. The effective enrichment of the activatable NIR-II fluorescent probe in target tissues is the fundamental guarantee of successful biological imaging. Therefore, drug delivery systems, including liposomes and mesoporous organosilicons, are used to deliver fluorescent probes to the target tissue and improve their concentration in the target tissue. (3) Safety. Although the activatable NIR-II fluorescent probes need special conditions to be activated, fluorescent probes in normal tissues will have toxic and side effects. Moreover, nano fluorescent probes with larger particle sizes are not easily cleared in vivo, and long-term toxicity arises. Therefore, research on safety should be enhanced to provide guarantees for clinical applications. 

In conclusion, activatable NIR-II fluorescent probes have undergone rapid development, which has effectively promoted research on fluorescence imaging. This review provides a reference for the design of, and research on, activatable NIR-II fluorescent probes and their applications in biological imaging.

## Figures and Tables

**Table 1:** Representatives of activatable NIR-II fluorescent probes for biomedical applications.

Activation Mode	Probe Name	Probe Fluorophores	Activation Mechanism	Excitation/ Emission (nm)	Biological Model	Refs
pH	BTC1070	Pentamethine cyanine	Protonation, inhibition of ICT effect	808/1000–1300	Simulated gastric fluid with different pH	[54]
pH	Ag_2_S Ve	Ag_2_S	Protonation, increased hydrophilicity	808/1250	4T1 breast cancer	[63]
β-Gal	BOD-M-βGal	BODPY derivatives	Hydrolyzation of β-galactose residues	808/900–1300	SKOV3 ovarian cancer	[72]
MMP14	A&MMP@Ag_2_S-AF7P	Ag_2_S	Consumption of NIR absorber A1094	785/1050	NB	[77]
H_2_O_2_	Ag/Ag_2_S JNP	Ag_2_S	Oxidizing the plasmonic Ag parts	825/1250	MCF-7 breast cancer	[82]
·OH	Hydro-1080	cyanine dyes	Conjugated system recovery	980/1100	Liver injury	[88]
HClO	SPNPs	PDF	Degradation of the receptor ITTC	808/1010	Inflamed paws	[95]
ONOO^−^	V&A@Ag_2_S	Ag_2_S	Consumption of A1094 chromophore	825/1050	TBI	[105]
ONOO^−^	V&C/PbS@Ag_2_Se	PbS@Ag_2_Se	Consumption of absorber Cy7.5	808/1616	Ischemic stroke	[109]
NO	AOSNP	Ag_2_S	Energy receptor units are converted	808/900–1150	Liver injury	[115]
H_2_S	WH-3	BODIPY derivatives	Release fluorescence quencher	980/1140	HCT-116 colon cancer	[121]
Hypoxia (NTR)	IR-1048-MZ	IR-1048	MZ group was reduced	980/1046	A549 lung cancer	[126]
Viscosity	WD-NO_2_	BODIPY derivatives	Intramolecular rotation of chemical bonds is limited	808/900–1400	Abnormal liver viscosity	[131]
Hyal and GSH	HISSNPs	IR-1061	Fracture of HA chains and disulfide bonds	808/1070	MCF-7 breast cancer	[139]

## Data Availability

Data are contained within the article.
